# Supplementary material for: How do farmland rental markets affect farmers’ income? Evidence from a matched renting-in and renting-out household survey in Northeast China
Source: PLoS One. 2021 Sep 30;16(9):e0256590. doi: 10.1371/journal.pone.0256590 (PMC8483330; doi:10.1371/journal.pone.0256590)
Supplement: Attachment — Submitted filename: Paper Review.docx [file pone.0256590.s003.docx]

**How do farmland rental markets affect farmers’ income? Evidence from a matched renting-in and renting-out households survey in Northeast China**

This paper addresses an important question: How do farmland rental markets affect farmers’ income? Evidence from a matched renting-in and renting-out households survey in Northeast China. The background to the study shows that farmland market renting is a very important issue in China where land is not owned by individual farmers. The authors present and discuss a good conceptual framework that leads to the use of a logit model to estimate the determinants of the rental market and the PSM models to measure the effect of the renting market on household income. The argument presented by authors to use this methodology is that it accounts for self-selection bias. They used data covering 810 rural households across 22 villages from Shandong province, which to them can be considered including a representative sample of participants of the rural land rental market in the targeted region.

The authors’ findings indicate that household off-farm income, family members’ part-time employment, and agricultural subsidies, agricultural cooperatives significantly affect farmers’ participation in the farmland rental market. Also, participation in the farmland rental market significantly increases the income of renting-in households, while it decreases the income of renting-out households which might result from the lag effect of the land system reform temporarily.

The flow of the paper making easy to read.

However, there are concerns that if addressed could improve the quality of the paper:

Data: The authors provided the reason why the two cities of Shandong province Dezhou and Qingdao were selected. However, the reasons why the selection of townships and villages differ between the two provinces are not given. Besides, nothing is said on how individual respondents were selected at the village level. There is the need to provide more light on the rationale in the distribution of the sample across participating and non-participating households on the one side, and across provinces and villages on the other side. Much more information is needed on the dataset – ie when the data were collected (ie, when during the agricultural season). Besides, describing how the outcome variables (income in this case and other key variables) were empirically measured is helpful particularly. We know that farmers in most of the cases have measurement problems, what effort was undertaken to reduce the measurement error on the outcome’s variables?

Participation: The definition of participation as given by the authors is clear enough. However, the definition is silent about the time frame that a household should participate to be considered as “Participant”. This is important for attribution.

Endogeneity. The current methodology used focuses on the selectivity bias. However, the authors raised the issue of bias due to unobservable factors. Some tests need to be performed to clarify whether or not these factors create the problem of endogeneity and if yes propose a way to deal with the problem.

Discussion and policy relevance: How do these results fit into the existing literature in this area? There was no effort by the authors to compare the results obtained in the study with the one in the literature. What is the policy relevance of this research? The current analysis supposes that participating in farmland renting market affect household the same way which is not the case in practice. It would be good to apply some decomposition perhaps by some socioeconomics characteristics such as gender to appreciate that difference.

Finally, the paper would benefit from some additional editing to ensure that concepts and terminology are stated clearly. Some of my edits are in the attachment.
