# Supplementary material for: How do farmland rental markets affect farmers’ income? Evidence from a matched renting-in and renting-out household survey in Northeast China
Source: PLoS One. 2021 Sep 30;16(9):e0256590. doi: 10.1371/journal.pone.0256590 (PMC8483330; doi:10.1371/journal.pone.0256590)
Supplement: Attachment — Submitted filename: (Ning Geng11.9)English paper_Gao.docx [file pone.0256590.s004.docx]

How do farmland rental markets affect farmers’ income? Evidence from a matched renting-in and renting-out households survey in Northeast China

**Ning Geng^1^, Zhifeng Gao^2^, Chuchu Sun^1^，Mengyao Wang^1^**

^1^ School of Public Administration, Shandong Normal University, Jinan, Shandong,250014, China; gengning@sdnu.edu.cn

^2^ Food and Resource Economics Department, University of Florida, Gainesville, Florida, 32608, America;

[zfgao@ufl.edu](mailto:zfgao@ufl.edu)

**Abstract:** Promoting farmland transfer through the farmland rental market is an essential instrument to achieve the scale economy of agricultural production in China. However, past literature on the land reform in China pays more attention to the renting-in household or the renting-out household respectively, less to both household together. Using a large-scale survey of farm households in China, we examine the determinants of participation in the farmland rental market and quantify the impact of rental market on farmers’ income. Findings show household off-farm income, family members’ part-time employment, and agricultural subsidies, agricultural cooperatives significantly affect farmers’ participation in the farmland rental market. Participation in the farmland rental market significantly increases the income of renting-in households, while it decreases the income of renting-out households which might result from the lag effect of the land system reform temporarily.

**KEYWORDS: l**and rental market；land property rights；income effect；propensity score matching method；China

## 1. Introduction

Land reforms implemented since the beginning of 1980s have greatly stimulated the development of rural land rental market in China. Since land is not privately owned in many countries, liberalizing rural land use rights has long been advocated as a good policy for agricultural economic growth and peasants’ well-being improvement in some developing countries. Starting 2002, there was an increased incidence of land rental activities in China (Shuyi Feng et al., 2010; Deininger & Jin, 2005). In the meanwhile, more and more rural young labors are pouring into China’s big cities to take up non-farm employment, accelerating the process of urbanization. Therefore, the development of rural land market is gaining momentum (Huang et al., 2012). The contribution to the economy growth of a well-functioning land rental market has well been recognized in China. Recently，the Chinese government further promote the development of rural land markets by means of “subcontacting, leasing, exchanging, or swapping” land-use rights (Huang et al., 2012). The land policy motivates farmers to rent more land to increase farm size and reduce rural land fragmentation ( Shi et al., 2017).

The development economics postulate that institutions play an essential role in facilitating economic growth (North, 1990). Securing property rights are expected to be an essential factor of an institutional environment contributing to development (de Soto, 2000). So the arguments over property rights have long been the root cause of land reform, more often because the vast majority of the rural population rely on the land to generate livelihoods (Migot-Adholla, 1991; Deininger and Feder, 2001; Fenske, 2010). In particular, the property rights reform of rural land resources is at the center of rural development and urbanization in contemporary China. As land is an essential factor of agricultural production, securing property rights is expected to not only improve productivity (Carol et al., 2015) but also motivate investment in agricultural production (Jerzy Michalek et al., 2014).

Land tenure security is an important precondition to developing a well-functioning rural land rental market (Besley, 1995). When rural land is rare, the certification of land use rights is expected to a crucial way to impart greater security (Hui W., 2015). The land-use rights certification can clearly identify the content of land use rights as well as confirm and secure the long-term land tenure. Some studies show that enhanced-tenure and land use rights certification promote participation in land rental market. Findings in China show that tenure-enhanced land reforms increased the land market activities by 5.5% from the renting-in households (Deininger et al., 2015). Studies also show that land certification can have positive effects on renting-out household but leave renting-in household unaffected (Deininger et al., 2011). In India, land certification induces greater engagement in the land rental market (Bardhan et al., 2014; Holden et al., 2011) and appears to have stimulated more land rental market activities in SNNP region in Ethiopia (Holden et al., 2016). Therefore improving tenure security and land certification is critical to the development of land rental market.

As rural land rental market activity has increased in recent decades, so have the number of studies estimating its effects on farmers. Regarding the impacts of rural land rental market, attention is mainly focused on efficiency, equity, and welfare. (1) It is well estimated that land rental market could have enhanced the allocative efficiency and agricultural productivity in rural China (Carter and Yao, 2002; Jin and Deininger 2009; Kimura et al, 2011). Research finds that land transfers from less efficient to more efficient farmers through land markets will help to reallocate land. Empirical evidence from southeastern China and Ethiopia suggested that rural land rental market could significantly boost economic growth and productivity, because renting-in households have a higher marginal product of rural land than renting-out households (Shuyi Feng, 2010; Klaus Deininger, 2013). (2) Regarding the effect on equity. Several recent studies concluded that rural land rental markets could promote equity in some developing countries by transferring land from land-rich farmers to land-poor farmers, and from labor-poor households to labor-rich household（Jacob Ricker-Gilbert, 2019; Chamberlin & Ricker-Gilbert, 2016; Yamano et al, 2009). Min Shi (2017) suggests that land rental market in China is a more effective method of rural land reallocation compared to the administrative distribution, without necessarily jeopardizing equity. (3) The welfare effect is primarily concerned with the increase in income of renting-in or renting-out land and the reduction of poverty. Land rental market can facilitate greater access to land for small farms as a primary productive asset (Songqing Jin & T.S. Jayne 2013). The empirical evidence from dynamic panel income models showed that the total income gains for the small farms from renting-in land were very remarkable (Klaus Deininger et al., 2013; Holden, Otsuka, and Place 2009). Renting-in land can not only increase the income of households but also reduce the incidence of family poverty (Chen & Zhai, 2015). The findings from Kenya show that renting-in land would result in an increase in per capita total income by 6.6% and a reduction in poverty status (Jin and Jayne 2013). Besides, off-farm employment also enables renting-out households to increase their earnings from both rental income and non-agricultural income (Feng & Heerink, 2008).

Despite the general finding of positive effects from rural land market, questions still remain about how the rental market improve income in the smallholder farming system for both renting-in and renting-out household. One of the major gap in the literature is that many researches neglect the income effect on renting-out households in the rental market as too much attention was paid to renting-in households. An exception is a study by Chen & Zhai (2015), which found that the net income effect for renting-in household mainly originates in the scale expansion of farmland and improvement of technology efficiency, while the income effect of renting-out household is mainly from off-farm income increase and partly from rent revenue.

In this study, we focus on the rental land market in the Shandong province that lies on the coast of northeast China and uses recently collected data on renting-in household and their matched renting-out household to estimate the income effect from land renting. Shandong is a noteworthy case in several aspects. First, Shandong is a province with better economic development where rapid changes in land transformation have taken place in the context of the development of industrialization and urbanization. Particularly the superior geographical location of the province provides many opportunities for farmers to engage in off-farm employment. Second, with a good natural condition, the province is a region of low flat land and an important grain production base. Thus, a well-functioning rental land market is crucial to ensure the nation’s grain supply. Third, Shandong province is representative with a high incidence of land transactions. Because of the above reasons, it will be interesting to estimate the income effect and the determinants of rural land rental market participation in Shandong province.

Our research is motivated by the two questions: (1) would the improving welfare attributed to land rental markets in the previous studies hold if renting-in and renting-out households are observed equally in the same dataset? (2) What are the determinants of farmers' participation in the land rental market?

We contribute to the current literature mainly from three aspects. First, we overcome the problem of estimating the income effect of renting-in and renting-out households separately. By compiling a dataset with both renting-in and renting-out households, we consider both the demand and supply sides of land rental market. Estimating the [economic](javascript:;) [benefit](javascript:;) to renting-in and renting-out households from land renting simultaneously, we can draw some conclusions that help make accurate land rental policies.

Second, we use the propensity score method (PSM) that can separate the effect of land renting from other factors that may affect the farm income. Thus the calculated results are relatively more convincing.

Third, we investigate the determinants that lead to farmers’ decisions on renting in or out rural land. Specifically, we determine whether the factors that affect households’ decision to rent in or out are different, and how the same factor may affect the participation in land rental market in different ways.

The rest of this article is organized as follows. In Section 2, we develop a conceptual framework to describe the determinants of participation in the land rental market. Section 3 mainly introduces the study site, the procedure of data collection, and descriptive statistics. Section 4 describes the empirical model to analyze the possibility that farmers will rent in or rent out land and the PSM method to measure the income effect of land rental market. In section 5, we discuss the estimation results of the models. Section 6 shows the summary and conclusions of our study.

## 2. Conceptual framework

Following an agricultural household model of production and rural land market participation (Deininger and Jin, 2005; Jin and Deininger, 2009), with the premise of “rational economic man”, we develop a conceptual model to determine the factors affecting a peasant’s participation in the rural land rental market.

Assume the household i choose the rural land to farm (Ai), the allocation of household labors engaged in the farming (Lia), the amount of labors for off-farm employment (Lio). We can use the following equation to model a household’s income maximization behavior:

$\mathrm{Max}{Income}_{i}=pf\left( a_{i},L_{ia},A_{i} \right)+wL_{io}-R_{i}^{in}[{(A}_{i}-\bar{A_{i}})\left( r+{MC}_{i}^{in} \right)]+R_{i}^{out}\left[ (\bar{A_{i}}-A_{i} )\left( r-{MC}_{i}^{out} \right) \right]$	(2.1)

$s.t. L_{ia}+L_{io}\leq\bar{L_{i}}$	(2.2)

In this equation, $\bar{L_{i}}$ and $\bar{A_{i}}$ are the fixed number of rural labor and land endowments, respectively, of the i household, while $a_{i}$is a set of household characteristics;$f\left( a_{i},L_{ia},A_{i} \right)$ is an agricultural production function; $p$ stands for the price of agricultural products;$w$ denotes the wage rate of off-farm employment for $L_{io}$; and $r$ is a competitive rent in the rental land market.$R_{i}^{in}$ and $R_{i}^{out}$are indicators standing for the renting-in ($R_{i}^{in}=1$ for renting-in land or 0 otherwise) and the renting-out ($R_{i}^{out}=1$ for renting-out land or 0 otherwise), respectively, of land. ${MC}_{i}^{in}$and ${MC}_{i}^{out}$ are the respective transaction costs for renting land in the land rental market.

Solve the income maximization problem, the following two equations define the conditions of the renting-in and renting-out of land:

$R_{i}^{in}=f \left( a_{i},{\bar{L_{i}},L}_{ia},L_{io},A_{i},\bar{A_{i}},{MC}_{i}^{in},{MC}_{i}^{out},w,r \right)$	(2.3)

$R_{i}^{out}=f \left( a_{i},{\bar{L_{i}},L}_{ia},L_{io},A_{i},\bar{A_{i}},{MC}_{i}^{in},{MC}_{i}^{out},w,r \right)$	(2.4)

Suppose $r$ is different for the local households due to the different plots of land. ${MC}_{i}^{in}$ and ${MC}_{i}^{out}$ are consistent for all farmers because they may face the risk of land loss, and the two indicators are related to the land tenure security (or certification of land renting contract), thus we can incorporate $r$, ${MC}_{i}^{in}, {MC}_{i}^{out}$, $A_{i},$ and $\bar{A_{i}}$ into the vector Mi which denotes rental market transaction variables. The labor variables (${\bar{L_{i}},L}_{ia},L_{io})$ and $a_{i}$can be represented by a vector of household characteristics (Hi), and Di is assumed to be family off-farm employment. Besides,$w$ can be included in the external environment (Wi). Hence we can derive the two reduced-form function associated with the equation 2.3 and 2.4 as follows:

$R_{i}^{in}=f \left( D_{i},H_{i},M_{i},W_{i} \right)$	(2.5)

$R_{i}^{out}=f \left( D_{i},H_{i},M_{i},W_{i} \right)$	(2.6)

The Eqs. (2.5) and (2.6) show that the determinants of renting-in or renting-out lands are determined by family off-farm employment (D), household characteristics (H), rental market transaction characteristics (M), as well as the external environment(W).

## 3. Survey and data

3.1 Study site

Shandong is an important agricultural province that lies on the east coast of China and across the sea from Japan and South Korea（Fig. 1, with a location suitable for an export-oriented economy. Shandong is also one of the most densely populated provinces of China, with small farms and high levels of land fragmentation cover approximately 157,100 km2. As the major grain-producing region, Shandong's grain production in 2017 was 53.2 million tons (Fig .2), accounting for 8 percent of the national total. Shandong led the country in total grain output value in 2017 (Fig. 3), reaching $60.635 billion. In sum, Shandong is a typical region that characterizes the plain area of northeast China and also a production base for ensuring food security.

Besides, Shandong has about 24.2 million rural laborers working off-farm by 2017. An efficient rural land market would not only help improve land productivity but also increases rural income. The Chinese government has promoted the reform of rural land property rights since 2015. The innovation of the land reform lies in liberalizing the land usage (or management) right^[1]^ and activating the land rental market according to supply and demand of farmland (Wang H, 2015), which can realize the scale economy of farmland and improve peasants’ income ( Vandeplas et al, 2013).

However, due to the influence of traditional Chinese thinking, in Shandong, many small-scale landholders are not willing to rent out their farmland, although these smallholders operate their land very inefficiently. In the meanwhile, productive farmers may not have the experiences to manage large-scale farmlands when they make a decision on renting in more land. Therefore, it is essential to determine the factors influencing farmers' participation in land rental market and whether participation in land rental market can result in the improvement of rural income.

**Fig.1** A map of the study area and sample distribution

Fig. 2. Gross grain output of China's provinces (top 9) in 2017 Unit: million tons Data source: China's National Bureau of Statistics (CNBS) 2017

|  |  |
| --- | --- |
| Heilongjiang | 75.1 |
| Henan | 66.5 |
| Shandong | 53.2 |
| Anhui | 40.1 |
| Hebei | 37 |
| Jiangsu | 36.6 |
| Jilin | 36.3 |
| Neimenggu | 35.5 |
| Sichuan | 34.9 |

Fig.3. Gross grain output value of China's provinces (top 9) in 2017 Unit: billion dollars Data source: China's National Bureau of Statistics (CNBS) 2017

|  |  |
| --- | --- |
| Shandong | 60.63 |
| Anhui | 41.55 |
| Jiangsu | 40.21 |
| Hubei | 36.15 |
| Guangdong | 33.01 |
| Henan | 28.94 |
| Sichuan | 27.67 |
| Hunan | 20.15 |
| Hebei | 15.66 |

3.2 Data collection

The data used in this article were collected from a socio-economic survey of landholders from December 2017 to November 2018 in Shandong. The survey collected detailed information on the socioeconomic characteristics of family members, [household](javascript:;) [income](javascript:;), farm, and off-farm activities, the amount of renting land, land-use history, the status of land certification, and so on (Min et al., 2017a).

To ensure that the sample includes typical renting-in and renting-off households, sample selection was carried out as follows. First, the survey was conducted in two cities of Shandong province Dezhou and Qingdao. Both regions are plain areas with large plots and have a more active land rental market in Shandong. More important, they are agricultural demonstration zones in Shandong, and more than 40% of farmland is traded in the land market.

Second, considering the matched data, sample areas are relatively large villages with more rural land to rent. Thus, four administrative townships were randomly selected from Dezhou city, and including three administrative villages in each sample township; while in Qingdao, we only selected ten administrative villages in two administrative sample townships.

Finally, to increase the response rate and obtain respondents' truthful opinions, we conducted the survey by face-to-face interviews. During the field survey, one enumerator took 1-2 hours to complete one questionnaire and each survey enumerator completed 3-5 questionnaires per day. Because our work focused on the land renting-in and renting-out behavior, when we interviewed the renting-out households, we should also find out the matching renting-in household to interview. To accurately estimate the income effect of land rental market, we also interviewed farmers who were not participating in the land rental market in the sample villages.

With the data covering 810 rural households across 22 villages from Shandong province, our study can be considered including a representative sample of participants of the rural land rental market. There are 403 non-participating households, 240 renting-out households, and 167 renting-in households (Table 1).

3.3 Definition and descriptive statistics

In this section, we define and describe the critical variable in the model that may affect farmers’ participation in the land rental market and their household income.

As discussed in Section 2, land rent in and rent out behaviors are influenced by four sets of variables such as household characteristics ($H_{i}$), family off-farm employment (Di), rental market transaction characteristics ($M_{i}$), as well as the external environment ($W_{i}$) (equations 2.3 and 2.4). The four dimensions and their relevant indicators (Table 1) are discussed below.

$H_{i}$ denotes a vector of household characteristics including age (“age”) and education (“edu”) of the householder. Generally, the average age of the renting-in group is significantly lower than that of the renting-out group, because more young households are more willing to rent in farmland for scale farming (Min et al., 2015). In addition, the renting-out farmers have a significantly higher education level, because well-educated farmers have more opportunities to find off-farm jobs.

Di is a vector of variables representing the number of farms and off-farm labors. In our study, we use the share of household off-farm income (“share”) and the ratio of family members` off-farm employment to farm employment (“parttime”) as [proxy variable](javascript:;)s of Di. As the rapid development of urbanization, the non-agricultural activities of ordinary farmers are gradually transferred to the service industries in the surrounding areas or urban areas. Therefore, the higher the proportion of non-agricultural income or the ratio of family members’ off-farm employment, the more farmers are inclined to rent out more farmland^[2]^.

The third variable dimension is $M_{i}$ including the rental market transaction variables, such as land operating area (area), a long-term certificate for land tenure (cont), the price of renting land (price), and the subsidies for production (subsidy). Land operating area is an important factor affecting rural land transfer as farmers with more land are more likely to rent land out (Huang and Ji, 2012). Additionally, the land price^[3]^ is a key element in the land rental market (Wang et al., 2011b). A high price of renting land may motivate more farmers to rent land out. Besides, long-term land tenure certificate is helpful to the development of land renting market. It has been [promulgate](javascript:;)d to peasants for ensuring land use right with a stable contract period since the “Rural Land Contract Law” was issued in China in 2002 (Huang and Ji, 2012; Deininger et al., 2014). At last, the subsidies for production (subsidy) will induce more demand for renting-in land because they can reduce the production cost when farm operation is expanded to a large production scale（Chen.F.,2015）.

$W_{i}$ is the fourth dimension denoting the external environment that may affect the land transfer. The variables included are the distance from the village to the urban area (“distance”), can be used to represent the off-farm opportunity information of household members. [Furthermore](javascript:;), participation in agricultural cooperatives benefiting their members is scale economies or the reduction of transaction costs to some extent ( Tafesse W. et al, 2019). So the control variable "cooper" has a significant impact on the renting-out and renting-in land. At present, in China, agricultural production has shifted from labor-intensive to technology-intensive production, so whether family members have received special technical training represents the technical level of farmers engaged in efficient agricultural production. The “tech” variable clearly indicates that farmers with more agricultural technologies are more inclined to rent in land to develop efficient agriculture.

| **Table 1.** Definition of relevant indicators of household`s livelihoods | | | |  |
| --- | --- | --- | --- | --- |
| **Category** | **Definition** | **Subitem** | **Abbreviation** | |
| *H_i_* | householder characteristics | 1.the age of householder | *age* | |
|  |  | 2.the sducation of householder | *edu* | |
| *D_i_* | family off-farm employment | 1.the share of household off-farm income | *share* | |
|  |  | 2. the ratio of family members` off-farm employment to farm employment | *parttime* | |
| *M_i_* | the land rental market ransaction | 1.the operating area of land | *area* | |
|  |  | 2.a long-term certificate for land tenure | *cont* | |
|  |  | 3.the price of renting land | *price* | |
|  |  | 4. the subsidies for production | *subsidy* | |
| *W_i_* | the external environment | 1. the distance from the village to the urban area | *distance* | |
|  |  | 2. the agricultural cooperative | *cooper* | |
|  |  | 3.agricultural technical training family members have received | *tech* | |

The statistical descriptions of all variables used in the regression are summarized in Table 2. The statistical results in table 2 show that the income level of the renting-in group was significantly higher than that of those not participating, and they passed the T-test at 1%, while the income level of the renting-out group was the lowest of the three groups. However, the statistical difference of the above indicators may not be the inevitable result of land renting, but caused by other factors. Therefore, we need to establish other econometric models to test the impact of land renting on household income.

| **Table 2.** Descriptive statistics of variables in the model | | | | | | | |
| --- | --- | --- | --- | --- | --- | --- | --- |
| **Variables** | | **Description** | **non-participants** | **rent-out group** | **Diff**  **B-A** | **rent-in group** | **Diff**  **C-A** |
|  |  |  | Mean  A | Mean B | T | Mean C | T |
| income | family per capita net income (Yuan) | | 7718.87 | 7075.54 | 0.94 | 73418.1 | -11.79^***^ |
| age | age | | 59.04 | 56.55 | 2.66^***^ | 47.27 | 12.31^***^ |
| edu | education | | 1.66 | 1.93 | 4.67^***^ | 2.54 | -13.97^***^ |
| share | the share of household non-farm income | | 0.52 | 0.42 | 2.91^***^ | 0.17 | 10.19^***^ |
| parttime | the ratio of family members` off-farm employment | | 1.39 | 1.85 | -7.16^***^ | 2.16 | -13.86^***^ |
| area | the operating aera of land (mu) | | 5.85 | 10.17 | -7.37^***^ | 189.84 | -17.74^***^ |
| price | land rent in rental market (Yuan per mu) | | 1276.96 | 1420.38 | -1.34 | 768.42 | 4.27^***^ |
| cont | =1,if have a land contract | | 1.13 | 1.2 | -2.54^**^ | 1 | 4.97^***^ |
| subsidy | the number of subsidies for production | | 1.01 | 1.3 | -8.54^***^ | 1 | 1.45 |
| tech | number of items gaining technical training | | 1.96 | 1.82 | 6.08^***^ | 1.02 | 57.03^***^ |
| cooper | =1,if join an agricultural cooperative | | 1.95 | 1.68 | 9.82^***^ | 1.77 | 6.66^***^ |
| distance | the distance from the village to the city | | 33.1 | 27.51 | 9.34^***^ | 29.14 | 5.74^***^ |

Note: *, **, and *** denote the significant coefficients at the levels of 10%,5%, and 1%, respectively.

4. Empirical Model

Following the above conceptual models, in this section, we will establish one economic model to estimate the determinants of farmers’ renting-in and renting-out land and use the propensity score matching (PSM) method to measure the income effect of land rental market.

### *4.1 Model specification*

To estimate the determinants of farmers’ renting-in or renting-out rural land by controlling independent variables ( as shown in Table 1), according to the Stochastic Utility Decision Model (Ali, A., and Abdulai, A., 2010; Ali, A., and Abdulai, A., 2010), we postulate U1^[4]^ and U0 representing the utility of farmers’ participation in the land rental market and non-participants, respectively. If V*= U1- U0>0, the farmers will choose to participate in the rental land market, otherwise not. Although we can’t observe V, it can be represented as a function of observable variables. We specify the following function models:

$V^{*}=f\left( X \right)+\mu, V=\left\{ \begin{aligned} 1 V^{*}>0 \\ 0 V^{*}<0 \end{aligned} \right.$	(4.1)

In the function (4.1), V is a two-value variable. If one household^[5]^ participate in the land rental market, V is equal to 1, otherwise, V is equal to 0. X is the vector of exogenous explanatory variables affecting farmers' participation in land rental market.$\mu$ is a [random](javascript:;) [disturbance](javascript:;) [term](javascript:;).

Furthermore, the detailed definitions and statistical descriptions of exogenous explanatory variables (X) are summarized in Table 1 and Table 2. So we can estimate the determinants of farmers’ renting-in and renting-out land by virtue of Logistic model (4.4).

$P \left( Y=1 | X \right)=F\left( X,\beta\right)=\varphi( X^{'}\beta)=\frac{exp(X^{'}\beta)}{1+exp(X^{'}\beta)}$	(4.2)

Suppose p=$P \left( Y=1 | X \right)=\frac{exp(X^{'}\beta)}{1+exp(X^{'}\beta)}$, and 1- p=$P \left( Y=0 | X \right)=\frac{1}{1+exp(X^{'}\beta)}$, we can get the following equation, $\frac{P}{1-P}=\exp\left( X^{'}\beta\right)$(4.3) Taking the log of both sides of Eq. 4.3, we get another equation as follows,

$ln(\frac{P}{1-P})=X^{'}\beta$	(4.4)

Thus, all the independent variables are in line with our conceptual models (2.5) and (2.6) as well as the econometric model (4.4).

### *4.2 Estimation approach*

Previous studies have shown that whether to participate in land rental market, farmers are "self-selecting", and some unobservable factors (such as production preference, management skills or family wealth, etc.) may affect the decision-making and lead to biased estimation results. [For](javascript:;) [this](javascript:;) [purpose](javascript:;), Rubin (1974) put forward “a counterfactual framework”, which was referred to as “Rubin Causal Model (RCM)”. The dichotomous Di=$\left\{ 0,\left. 1 \right\} \right.$ indicates whether the household ith participate in the land rental market. [Correspondingly](javascript:;), the counterfactual outcomes are y1i and y0i, respectively, participation and non-participation in the land rental market.

$y_{i}=\left\{ \begin{aligned} y_{1i} ifV_{i}=1 \\ y_{0i} if V_{i}=0 \end{aligned} \right.$	(4.5)

The treatment effect for i is thereby ( y1i- y0i), this is what we want to verify. y1i and y0i can’t be observed at the same time. So we can model the household i’s observed outcome as follows:

$y_{i}=\left( 1-V_{i} \right)y_{0i}+V_{i}y_{1i}=y_{0i}+V_{i} ( y_{1i}- y_{0i})$	(4.6)

In Eq.(4.5), $y_{i}$is impacted by the household i’s traits set $X_{i}$, the exogenous explanatory variables set $X_{j}$ and the random unobservables $\mu_{i}$. For the same household i, $\mu_{i}$ represents the unobserved determinants of outcomes following a joint normal distribution, such as the household’s production preferences, managerial skills, or household wealth. On this basis, the influence equation of participating in farmland capitalization on farmers' welfare can be set as follows:

$$y_{i}=f_{0}\left( X_{i},X_{j} \right)+\mu_{0i}+V_{i}\left[ {(f}_{1}\left( X_{i},X_{j} \right)+\mu_{1i})-{(f}_{0}\left( X_{i},X_{j} \right)+\mu_{0i}) \right]$$

$=f_{0}\left( X_{i},X_{j} \right)+V_{i}\left[ f_{1}\left( X_{i},X_{j} \right)-f_{0}\left( X_{i},X_{j} \right)+(\mu_{1i}-\mu_{0i}) \right]+\mu_{0i}$	(4.7)

Moreover, whether the household ith participate in land capitalization does not affect other individuals, which is deemed to “stable unit treatment value assumption” (SUTVA) which excludes inter-individual social interactions or general equilibrium. The treatment effect (y1i- y0i) is a [random](javascript:;) [variable](javascript:;), so we just [pay](javascript:;) [attention](javascript:;) [to](javascript:;) the expected value E.

To solve "self-selection" of research objects and the influence of unobservable factors (such as production preference, management skills or family wealth, etc.), this article adopted the propensity score matching (PSM) method and selected farmers (control group) who did not participate in the capitalization for the matching analysis. Rosenbaum ＆ Rubin(1985) constructed a counterfactual analysis framework different from Eq. (4.7) so as to effectively eliminate the biased estimation of non-random distribution of samples. We need to find a control group as similar to the treat group to reduce the bias of sample selection. For those having participated in land capitalization, we estimate the average treatment-on-the-treated effect (ATT) by:

$$ATT=E\left( y_{1i} | X_{i},X_{j},D_{i}=1 \right)-E\left( y_{0i} | X_{i},X_{j},D_{i}=1 \right)$$

$=E\left( y_{1i}-y_{0i} | X_{i},X_{j},D_{i}=1 \right)$	(4.8)

Eq.(4.8) shows that$E\left( y_{1i} | X_{i},X_{j},D_{i}=1 \right)$ is observable, while $E\left( y_{0i} | X_{i},X_{j},D_{i}=1 \right)$ is unobservable that's called a counterfactual result. Therefore, an alternative indicator $E\left( y_{0i} | X_{i},X_{j},D_{i}=1 \right)$can be constructed using the propensity score matching method (PSM). On the premise that the covariates$\left( X_{i},X_{j} \right)$ of the two groups are as similar or same as possible, the propensity score of each sample to enter the treatment group is calculated. This method can not only accurately evaluate the income effect of the land rental market, but also compare the source and difference characteristics of the income effect of two groups of matched samples.

5. Estimation results and discussion

5.1 Determinants of farmers' participation in land rental market

Before the regression estimation, we firstly analyzes the correlation of the covariates variables X in the included model and tests the possible multicollinearity problems. Using variance inflation factor (VIF) and tolerance (TOL) to validate the results show that (table 3), two groups of each control variable average variance inflation factor of 1.24 and 1.45 respectively, and the biggest VIF in each control variable has a value of 2.58, the lowest value of TOL also reached 0.39. Therefore, it can be judged that the multicollinearity problem in the included model is not serious and the model is within an acceptable range.

| **Table 3.** Measurement results of variance inflation factor and tolerance of each variable | | | | |
| --- | --- | --- | --- | --- |
| **Variable** | **rent-out** | | **rent-in** | |
|  | **VIF** | **TOL** | **VIF** | **TOL** |
| age | 1.4 | 0.72 | 1.53 | 0.65 |
| edu | 1.42 | 0.7 | 1.64 | 0.61 |
| share | 1.32 | 0.76 | 1.53 | 0.66 |
| parttime | 1.22 | 0.82 | 1.63 | 0.62 |
| area | 1.3 | 0.77 | 1.74 | 0.57 |
| price | 1.16 | 0.86 | 1.13 | 0.88 |
| cont | 1.14 | 0.87 | 1.08 | 0.92 |
| subsidy | 1.14 | 0.88 | 1.02 | 0.98 |
| tech | 1.3 | 0.77 | 2.58 | 0.39 |
| cooper | 1.11 | 0.89 | 1.27 | 0.79 |
| distance | 1.29 | 0.78 | 1.23 | 0.81 |
| Mean VIF | 1.24 |  | 1.45 |  |

To further measure the income effect of land rental market on different sample households, and to provide conditional probabilistic fitting values for calculating the household income effects (ATT), a flexible logit model was used for regression estimation. We first estimate two functions as Eqs.(2.5) and (2.6). The regression results are reported in Table 4. Furthermore, the following conclusions can be drawn from the coefficient estimation results of the key variables in the two models.

First, in the model for renting-in land, the correlated coefficient of independent variable “age”, “share”, “parttime” ,“price”, “subsidy”, “tech”, “cooper” is remarkable at the levels of 1% and 10%, respectively. Table 2 shows that the average age of the renting-in group is significantly lower than that of the renting-out group. The results in Table 4 presented that relatively young households are more willing to rent in farmland for scale farming. Moreover, the explanatory variable “share” and “parttime” denote the off-farm employment of household members, due to the low comparative returns of agriculture, the non-agricultural production activities of ordinary farmers are gradually transferred to the primary service industries in the surrounding areas and urban areas. Therefore, the higher the proportion of non-agricultural income and the level of off-farm employment of family members, the more inclined farmers are to rent in farmland^[6]^. Table 4 also shows that the other explanatory variables, e.g., the price of renting land, the subsidies for production, the agricultural cooperative and agricultural technical training, significantly affect farmers’ participation in the land rental market. In line with our theoretical analysis, land price^[7]^ is a key element of the land rental market. If it is farmers' "self-selection" behavior, the higher the land rent is, the less likely it is to rent in the land. At present, agricultural production has shifted from labor-intensive to technology-intensive production, so whether family members have received special technical training represents the technical level of farmers engaged in efficient agricultural production. The “tech” variable clearly indicates that farmers are more inclined to rent in land to develop efficient agriculture. Besides, the control variable "cooper" has a significant impact on the renting-out and renting-in land. This is closely related to the reduction of transaction costs by joining cooperatives.

Second, in the model for renting-out land, the independent variable “edu” denotes that a higher level of landholder’s education will increase the likelihood of renting out land and then work in off-farm employment. Moreover, The other variables, e.g., “share”, “parttime”, “subsidy” and “cooper” have the same impact on renting out land as in the model for renting-in land. This is an indication that the above factors influence the participation of farmers in the land rental market. Furthermore, the index of land operating area (area) has also passed the significance test and is positively correlated with renting out land. Households with more land are more probable to rent out land. This might be because land rich households have more chances to earn money instead of specializing in farming. Finally, the distance from the village to the urban area can be used to represent the opportunity information of farmers to go out for work. The closer it is to the urban area, the more off-farm employment opportunities farmers will have, and the more willing they are to rent out their land.

| **Table 4.** Results of Logit model: determinants of farmers' participation in land rental market | | | | |
| --- | --- | --- | --- | --- |
| **variables** | **renting-out** | | **renting-in** | |
|  | **Coefficient** | **Std.error** | **Coefficient** | **Std.error** |
| Constant | 5.11 | 2.01 | 27.47^***^ | 8.424 |
| age | -0.001 | 0.012 | -0.269^***^ | 0.071 |
| edu | 0.418^**^ | 0.191 | 0.261 | 0.917 |
| share | -1.388^***^ | 0.363 | -9.583^***^ | 2.762 |
| parttime | 1.311^***^ | 0.19 | 4.934^***^ | 1.285 |
| area | 0.084^***^ | 0.027 |  |  |
| price | 0.0003 | 0.0009 | -0.001^***^ | 0.003 |
| cont | 0.295 | 0.355 |  |  |
| subsidy | 2.518^***^ | 0.313 | 4.135^***^ | 0.232 |
| tech | -0.334 | -0.498 | -6.741^***^ | 1.289 |
| cooper | -4.051^***^ | 0.478 | -4.316^*^ | 2.619 |
| distance | -0.109^***^ | 0.019 | -0.044 | 0.065 |
| R-squared | 0.441 |  | 0.92 |  |
| Log likelihood | -237.39 |  | -25.26 |  |
| Number of oservations | 641 |  | 570 |  |

Note: *, **, and *** denote the significant coefficients at the levels of 10%,5%, and 1%, respectively.

5.2 Measurement and analysis on income effect of land rental market

The most important purpose of propensity score estimation is to balance the distribution of variables between experimental and treatment groups rather than to obtain accurate probabilistic predictions. Therefore, to minimize the inter-group differences in the probability distribution of sample households of different groups and ensure high-quality matching results, this article carried out a balance test on explanatory variables and control variables of the experimental group and the treatment group before the PSM model estimation. Table 5 shows that the results of the contrast before the match, the standard deviation (% bias) of [explanatory](javascript:;) and most control variables after the match have shrunk to less than 10%, and most of the T-test result is significant. Especially important, it can not only largely reduce the total matching bias, but also can effectively reduce the differences between groups of different samples. The standardized deviation of individual control variables is more than 10% after matching, but still within the acceptable range. The above test results show that the matching of explanatory variables and control variables between different samples is desirable, and it has certain persuasive power to explain the change of farmers' welfare level.

| **Table 5** .Balance test of control variables by nearest neighbor matching method | | | | | | | |
| --- | --- | --- | --- | --- | --- | --- | --- |
| variables | unmatched  matched | renting -out | non-  participants | bias  % | renting  -in | non-  participants | bias  % |
|  |  | Mean | |  | Mean | |  |
| age | U | 56.93 | 58.91 | -17.5 | 52.21 | 58.91 | -31.2 |
|  | M | 56.84 | 56.61 | 1.6 | 53.42 | 56.61 | 6.4 |
| edu | U | 1.93 | 1.67 | 33.2 | 2.54 | 1.68 | 122.2 |
|  | M | 1.906 | 2.0573 | -19.1 | 2.33 | 2.36 | -5.1 |
| rate | U | 0.48 | 0.53 | 14.6 | 0.32 | 0.53 | -53.8 |
|  | M | 0.52 | 0.47 | 13.5 | 0.36 | 0.47 | -27.6 |
| parttime | U | 1.88 | 1.43 | 11.7 | 2.01 | 1.43 | 2.01 |
|  | M | 1.74 | 1.78 | -3.9 | 1.78 | 1.78 | 1.78 |
| area | U | 9.57 | 6.05 | 44 | 89.84 | 6.05 | 75.6 |
|  | M | 7.25 | 9.54 | -28.6 | 54.97 | 9.54 | 40 |
| tech | U | 1.84 | 1.96 | 13.9 | 1.44 | 1.96 | -37.8 |
|  | M | 1.89 | 1.74 | 6.5 | 1.52 | 1.74 | 24.9 |
| price | U | 1447.2 | 1366.3 | 5.6 | 1115.8 | 1366.3 | -17.1 |
|  | M | 1536.6 | 1677.7 | -9.8 | 1204.8 | 1677.7 | -12.4 |
| cont | U | 1.24 | 1.12 | 30.2 | 1.12 | 1.12 | 14.4 |
|  | M | 1.16 | 1.12 | 10.9 | 1.09 | 1.12 | -2.5 |
| cooper | U | 1.71 | 1.93 | -61.5 | 1.74 | 1.93 | -56 |
|  | M | 1.76 | 1.98 | -60.4 | 1.88 | 1.98 | -9.1 |
| distance | U | 26.79 | 32.75 | -91.2 | 29.14 | 33.15 | -53.3 |
|  | M | 27.38 | 32.11 | -72.4 | 28.4 | 28.89 | -6.5 |

After obtaining effective matching samples, we calculated the income effect of land rental market (ATT) as in Eq.(4.8). When Stata12.0 software was used for the actual matching measurement since the selected sample individuals (control group) in this study were limited, to reduce the loss of effective samples, the matching was carried out with a replacement, and different matching methods were used for the measurement. The results in table 6 show that, from a quantitative perspective, although the values of the four matching results are different, the numerical directions are consistent, and most of them have passed the significance test. From the comparative analysis of the two main welfare effects, the welfare effect of renting-out farmland is negative while renting-in farmland has a significant increase in income. In other words, the average per capita net income of a renting-out household will [decrease](javascript:;) by 567.29 yuan, while the average per capita net income of a renting-in household will increase by 26386.53 yuan. Aslo, by comparing the characteristics of descriptive indicators table 1 can be found, based on the tendency of scoring method to measure the statistical summary of the results, much lower than visible tend to score method to land capitalization independently from other factors that affect farmers welfare effect, to examine the land capitalization this explanation variable for the effect of farmers' welfare level, and the calculation results are relatively more convincing. More importantly, considering the limitations of PSM, the implicit bias caused by unmeasurable factors should be reduced, that is, the influence of the implicit bias on the matching results should be judged to be serious. This article uses the "Rosenbaum Bounds" rule to test the sensitivity of the matching results (Rosenbaum,1985). Test results show that when Γ = 1.5, corresponding to the P-value is still significant at 5% and 10% levels, an observation of the visible research samples influence factors on the controlled variable estimates the ATT value is relatively small and estimates the results more credible.

| **Table 6.** The net income effect of rural land rental market | | | | | | | |
| --- | --- | --- | --- | --- | --- | --- | --- |
| Matching methods | welfare indicator | renting-out welfare effect | | | renting-in welfare effect | | |
|  |  | ATT | T | Sensitivity test（Γ=1.5）：sig+ | ATT | T | Sensitivity test（Γ=1.5）：sig+ |
| neighbor mat （1-5） | Family per capita net income | -632.46 | -0.4 | 0.065 | 25080.69 | 2.38 | 0.0214 |
| neighbor mat  (1-10) |  | -144.79 | -2.53 | 0.056 | 25788.68 | 2.45 | 0.0205 |
| kernel mat |  | -451.16 | -1.78 | 0.048 | 24265.97 | 1.61 | 0.0413 |
| radius mat |  | -1040.73 | -2.27 | 0.0534 | 30410.77 | 2.93 | 0.0527 |
| Mean |  | -567.29 |  |  | 26386.53 |  |  |

Based on the above regression results (Table 4) and the calculated results of the income effect of farmers (Table 6), the following analysis can be made. Firstly, land rental market can significantly increase the income level of the renting-in household. This is mainly related to the government's financial subsidies for production. However, the income level of renting-out households is declining.

Secondly, for the renting-out household, “education”、“the share of household non-farm income（share）”、“the ratio of family members` part-time employment （parttime）”、“household contracted farmland area (area)”、“whether to join agricultural cooperatives （copper）” and “the distance from the village to the city (distance) are the key elements for renting-out household. [Theoretically](javascript:;) [speaking](javascript:;), only the total income including land rent and non-agricultural income is much higher than the net income of agricultural production, farmers are willing to rent out their land voluntarily. However, the income level of renting-out households has not been improved significantly maybe [on](javascript:;) [account](javascript:;) [of](javascript:;) the lag effect of the land system reform.

Thirdly, for the renting-in household^[8]^, The results show that reducing production cost (“subsidy”) and improving technology level (“tech”) are the key influencing factors of current land scale management households. The variables of “age” indicate the ability of farmers to operate and manage the land, while the scientific and technological level of farmers and the number of agricultural subsidies respectively determine the output boundary of land managers and the profit space of scale operations.

6. Conclusion

This study has evaluated the income effect of farmers participating in land rental market and analyzed farmers’ behavior relevant to participation in the land rental market. Taking comprehensive account of four dimensions of livelihoods decision-making of farmers, we find that to an average-level rural [household](javascript:;)s, land rental market evidently increased the level of income for farmers who rented-in farmland while reducing the level of income for farmers who rented out their land. Two reasons may contribute to the result. Firstly, given the long-term goal of gaining scale benefits, up-to-bottom land reform by the government policy-maker brings about more support for renting-in rural households to realize scale-operation of farmlands, including some [agricultural](javascript:;) [subsidies for production.](javascript:;) For the other hand, since our data only covers the initial third year of the land reform, the lag effect of the land system reform may result in a decline in the level of income for renting-out rural households. In addition, the [potential](javascript:;) reason may also be consistent with the phenomenon of abandoned land.

Some caution is needed finally. First, although the income level of renting-out households has declined, it does not mean that China's land reform is not successful. Second, during the initial some years of the land reform, the increase in transaction costs may be the short-term frictional institutional cost. Third, the data in this study are from the land reform demonstration zones. Subsequent studies will expand the sample areas with different levels of economic development and carry out research based on larger sample data.

**Author Contributions:** For research articles with several authors, their contributions are as follows: “Writing- original draft, Ning Geng; Writing- reviewing & editing, Zhifeng Gao ; Data collecting, Chuchu Sun; Investing, Mengyao Wang; All authors have read and agreed to the published version of the manuscript.

**Funding:** This research was funded by “the ministry of education and social science youth program, grant number 17YJC790041” ,“The national natural science foundation of China program, grant number 71803104” and Qingchuang Science and Technology Support Plan for Colleges and Universities in Shandong Province ( grant number 2019RWE009) .

# References

1. Ali A, Abdulai A. The Adoption of Genetically Modified Cotton and Poverty Reduction in Pakistan. *Journal of Agricultural Economics*. 2010;61(1):175-92.

2. Besley, T. Property rights and investment incentives: Theory and evidence from Ghana. *Journal of Political Economy*, 1995;103(5), 903–937.

3. Bardhan P, Luca M, Mookherjee D, Pino F. Evolution of land distribution in West Bengal 1967-2004: Role of land reform and demographic changes. *Journal of Development Economics*. 2014;110:171-90.

4. Becerril J, Abdulai A. The Impact of Improved Maize Varieties on Poverty in Mexico: A Propensity Score-Matching Approach. *World Development*. 2010;38(7):1024-35.

5. Carol Newman, Finn Tarp, and Katleen van den Broeck, Property Rights and Productivity: The Case of Joint Land Titling in Vietnam. *Land Economics*. 2015;91(1):91-105.

6. Carter MR, Yao Y. Local versus global separability in agricultural household models: The factor price equalization effect of land transfer rights. *American Journal of Agricultural Economics*. 2002;84(3):702-15.

7. Chamberlin J, Ricker-Gilbert J. Participation in rural land rental markets in sub-Saharan Africa: who benefits and by how much? evidence from Malawi and Zambia. American *Journal of Agricultural Economics*. 2016;98(5):1507-28.

8. Chen F., Zhai W. J. Land transfer incentive and welfare effect research from perspective of farmers’ behavior. *Economic Research*, 2015;（10）：163-177. (In Chinese)

9. Deininger K, Jin SQ. The potential of land rental markets in the process of economic development: Evidence from China. *Journal of Development Economics.* 2005;78(1):241-70.

10.De Soto, H. The mystery of capital: Why capitalism triumphs in the West and fails everywhere else. *New York: Basic Books*. , 2000.

11.Deininger K, Jin S, Liu S, et al. Impact of property rights reform to support China's rural-urban integration: household-level evidence from the Chengdu National Experiment. *World Bank Policy Research Working Paper* 7388, 2015.

12.Deininger K, Ali DA, Alemu T. Impacts of Land Certification on Tenure Security, Investment, and Land Market Participation: Evidence from Ethiopia. *Land Economics*. 2011;87(2):312-34.

13.Deininger, K., Jin, S. The potential of land rental markets in the process of economic development: evidence from China. *Journal of Development Economics.* 2005 ;78 (1), 241–270.

14.Deininger K, Jin S, Xia F. Moving Off the Farm: Land Institutions to Facilitate Structural Transformation and Agricultural Productivity Growth in China. *World Development*. 2014;59:505-20.

15.Feng S, Heerink N, Ruben R, Qu F. Land rental market, off-farm employment and agricultural production in Southeast China: A plot-level case study. China Economic Review. 2010;21(4):598-606.

16.Feng S, Heerink N. Are farm households' land renting and migration decisions inter-related in rural China? Njas-Wageningen. *Journal of Life Sciences*. 2008;55(4):345-62.

17.Huang J, Gao L, Rozelle S. The effect of off-farm employment on the decisions of households to rent out and rent in cultivated land in China. *China Agricultural Economic Review.* 2012;4(1):5-17.

18.Wang H, Riedinger J, Jin S. Land documents, tenure security and land rental development: Panel evidence from China. *China Economic Review.* 2015;36:220-35.

19.Holden ST, Deininger K, Ghebru H. Tenure Insecurity, Gender, Low-cost Land Certification and Land Rental Market Participation in Ethiopia. *Journal of Development Studies.* 2011;47(1):31-47.

20.Holden S T, Bezu S, Tilahun M. How pro-poor are land rental markets in Ethiopia?.*Centre for Land Tenure Studies Report,* March. 2016

21.Huang, J., Gao, L., Rozelle, S. The effect of off-farm employment on the decisions of households to rent out and rent in cultivated land in China. *China Agricultural Economics.* 2012;Rev.4 (1), 5–17.

22.Holden, S.T., K. Otsuka, and F.M. Place. “Understanding Land Markets.”In The Emergence of Land Markets in Africa: Impacts on Poverty, Equity and Efficiency, Chapter 2, ed. S.T. Holden, K.Otsuka, and F.M. Place, 18–51.*Washington DC: Resources for the Future Press.* 2009b.

23.Huang, J., Gao, L., Rozelle, S. The effect of off-farm employment on the decisions of households to rent out and rent in cultivated land in China. *China Agricultural Economics.* 2012;Rev.4 (1), 5–17.

24.Jerzy Michalek, Pavel Ciaian, and d’Artis Kancs. Capitalization of the single payment scheme into land value: Generalized Propensity Score Evidence from the European Union. *Land Economics.* 2014;90 (2),260–289.

25.Jin S, Deininger K. Land rental markets in the process of rural structural transformation: Productivity and equity impacts from China. *Journal of Comparative Economics.* 2009;37(4):629-46.

26.Jacob Ricker-Gilbert, Jordan Chamberlin, Joseph Kanyamuka, Charles B.L. Jumbe, Rodney Lunduka, Stevier Kaiyatsa. How do informal farmland rental markets affect smallholders’well-being? Evidence from a matched tenant–landlord survey in Malawi. *Agricultural Economics.* 2019;50(5):595–613.

27.Kimura S, Otsuka K, Sonobe T, Rozelle S. Efficiency of Land Allocation through Tenancy Markets: Evidence from China. *Economic Development and Cultural Change*. 2011;59(3):485-510.

28.Klaus Deininger , Daniel Ayalew Ali & Tekie Alemu, Productivity effects of land rental market operation in Ethiopia: evidence from a matched tenant–landlord sample. *Applied Economics*, 2013;.45, 3531–3551.

29.Kimura S, Otsuka K, Sonobe T, Rozelle S. Efficiency of Land Allocation through Tenancy Markets: Evidence from China. *Economic Development and Cultural Change.* 2011;59(3):485-510.

30.Min S, Bai J-f, Seale J, Jr., Wahl T. Demographics, societal aging, and meat consumption in China. *Journal of Integrative Agriculture.* 2015;14(6):995-1007.

31.North, D. C. Institutions, institutional change, and economic performance. *New York: Cambridge University Press*. 1990.

32.Rubin, D.B. Estimating causal effects of treatments in randomized and nonrandomized studies, *Journal of Edeucational Psychology,* 1974;.66:688-701.

33.Rosenbaum, P.R. and D.B.Rubin.Coustructing a control group using multivariate matched sampling methods that incorporate the propensity score.*The American Statistician,* 1985;39(1):33-38.

34.Shuyi F. , Nico Heerink, Ruerd Ruben, Futian QU. Land rental market, off-farm employment and agricultural production in Southeast China: A plot-level case study. *China Economic Review,*2010;21,598-606.

35.Min S, Waibel H, Huang J. Smallholder participation in the land rental market in a mountainous region of Southern China: Impact of population aging, land tenure security and ethnicity. *Land Use Policy.* 2017;68:625-37.

36.Songqing Jin & T.S.Jayne. Land Rental Markets in Kenya: Implications for efficiency, equity, household income, and Poverty. *Land Economics,* 2013;89 (2): 246–271.

37.Tafesse W. Gezahegn , Steven Van Passel,Tekeste Berhanu, Marijke D’Haese,Miet Maertens. Big is efficient: Evidence from agricultural cooperatives in Ethiopia. *Agricultural Economics,*2019 ;50(7):555–566.

38.Vandeplas, A., Minten, B., & Swinnen, J. Multinationals vs. cooperatives: the income and efficiency effects of supply chain governance in India. *Journal of Agricultural Economics*, 2013;64, 217–244.

39.Wang H, Riedinger J, Jin S, Land documents, tenure security and land rental development: Panel evidence from China. *China Economic Review,* 2015 ;36: 220-235.

40.Wang H, Tong J, Su F, Wei G, Tao R. To reallocate or not: Reconsidering the dilemma in China's agricultural land tenure policy. *Land Use Policy.* 2011;28(4):805-14.

41.Yamano, T., Place, F. M., Nyangena, W., Wanjiku, J., & Otsuka, K. Chapter 5: Efficiency and equity impacts of land markets in Kenya. In S. T. Holden, K. Otsuka, & F. M. Place (Eds.), The emergence of land markets in Africa: Impacts on poverty, equity and efficiency (pp. 93–111). *Washington, DC: Resources for the Future Press*. 2009.

1. In China, rural land ownership belong to the rural collective organization, farmers only have contracted management and use rights.
2. in today's China, renting a lot of lands requires hiring labor. so hiring labor can replace family members.
3. In this paper, land price is mainly represented by land rent.
4. the farmers who participate in the rural land rental market include both the renting-in and renting-out household.
5. The household who may rent in land or rent out land is described as the participants in the rental land market.
6. in today's China, renting a lot of lands requires hiring labor. so hiring labor can replace family members.
7. In this paper, land price is mainly represented by land rent.
8. the research objects for renting-in households in this paper are mainly family farms, professional large family.
