# Supplementary material for: How do farmland rental markets affect farmers’ income? Evidence from a matched renting-in and renting-out household survey in Northeast China
Source: PLoS One. 2021 Sep 30;16(9):e0256590. doi: 10.1371/journal.pone.0256590 (PMC8483330; doi:10.1371/journal.pone.0256590)
Supplement: Attachment — Submitted filename: Response to reviewer 2.docx [file pone.0256590.s005.docx]

**Reviewer #2:**

This paper addresses an important question: How do farmland rental markets affect farmers’ income? Evidence from a matched renting-in and renting-out households survey in Northeast China. The background to the study shows that farmland market renting is a very important issue in China where land is not owned by individual farmers. The authors present and discuss a good conceptual framework that leads to the use of a logit model to estimate the determinants of the rental market and the PSM models to measure the effect of the renting market on household income. The argument presented by authors to use this methodology is that it accounts for self-selection bias. They used data covering 810 rural households across 22 villages from Shandong province, which to them can be considered including a representative sample of participants of the rural land rental market in the targeted region.

The authors’ findings indicate that household off-farm income, family members’ part-time employment, and agricultural subsidies, agricultural cooperatives significantly affect farmers’ participation in the farmland rental market. Also, participation in the farmland rental market significantly increases the income of renting-in households, while it decreases the income of renting-out households which might result from the lag effect of the land system reform temporarily.The flow of the paper making easy to read.

**Reply:**

Thank you for the comments. We have addressed the issues that you raised below item by item. We have added new figures, running some new analyses to address your concerns. In the places where no revisions are done, we carefully explained what we have done and why to choose what we have done. We hope the revised manuscript adequately addressed all your concerns.

However, there are concerns that if addressed could improve the quality of the paper:

**Data: The authors provided the reason why the two cities of Shandong province Dezhou and Qingdao were selected. However, the** **reasons why the selection of townships and villages differ between the two provinces are not given. Besides, nothing is said on how individual respondents were selected at the village level. There is the need to provide more light on the rationale in the distribution of the sample across participating and non-participating households on the one side, and across provinces and villages on the other side. Much more information is needed on the dataset – ie when the data were collected (ie, when during the agricultural season). Besides, describing how the outcome variables (income in this case and other key variables) were empirically measured is helpful particularly. We know that farmers in most of the cases have measurement problems, what effort was undertaken to reduce the measurement error on the outcome’s variables?**

**Reply:** Thanks for the comment. Please see our reply to your comment below.

First, regarding the comments “reasons why the selection of townships and villages differ between the two provinces are not given.”

“Shandong is one of the most densely populated provinces of China, with small farms and high levels of land fragmentation cover approximately 157,100 km2. As the major grain-producing region, Shandong's grain production in 2017 was 53.2 million tons (Fig 2), accounting for 8 percent of the national total. Shandong led the country in total grain output value in 2017 (Fig 3), reaching $60.635 billion.” (Page 8). We select Dezhou and Qingdao of Shandong province, “because they are agricultural demonstration zones in Shandong, and more than 40% of farmland is traded in the land market.” And one of the key difference between the two cities is that “the proportion of traded land in Qingdao is approximate 50%, while Dezhou trades 42%.”(Page 8)

**Second,** regarding “nothing is said on how individual respondents were selected at the village level”,

We added, “we randomly selected respondents in the sample villages” in the revised manuscript (page 10).

**Third,** regarding the comment “There is the need to provide more light on the rationale in the distribution of the sample across participating and non-participating households on the one side, and across provinces and villages on the other side.”

Among the total 810 rural households interviewed, there are 407 participating households, including 240 renting-out households and 167 renting-in households. The specific distribution of these households by the city is that there are 559 rural households interviewed in Qingdao, with 266 participating households that include 165 renting-out households and 101 renting-in households. In comparison, there are 251 rural households interviewed in Dezhou, with 141 participating households that include 75 renting-out households and 66 renting-in households. The samples are distributed in such a way mainly because the population in Qingdao is much larger than that of Dezhou. In the manuscript, we did not provide detailed distribution of participating vs. non-participating households by city because our study does not try to determine the welfare effect of land market participation by city. In addition, it is not possible to conduct PSM analysis if we separate the sample by city because of the smaller sample size for each city.

**Fourth,** regarding the comment that “Much more information is needed on the dataset – ie when the data were collected (ie, when during the agricultural season).”

We randomly selected respondents in the sample villages during the non-farming season because most farmers are busy with agricultural production and do not have much time to participate in the survey during the farming season. On page 10, we stated that “The data used in this article were collected from a socio-economic survey of landholders from December 2017 to November 2018 during the non-farming season in Shandong.”

**At last,** regarding the comment that “Besides, describing how the outcome variables (income in this case and other key variables) were empirically measured is helpful particularly. We know that farmers in most of the cases have measurement problems, what effort was undertaken to reduce the measurement error on the outcome’s variables?”

In our study, income is measured by asking the interviewee “[household](javascript:;) [incomes](javascript:;) [per](javascript:;) [capita](javascript:;) ”. Several methods are used to reduce the measurement errors of the outcome variable. For instance, face-to-face interviews were used to increase the trust between the interviewer and interviewee, therefore increasing the response rate and obtain respondents' truthful opinions. Before the survey was carried out, we also made it clear to the interviewees in advance that our survey was only for scientific research and had no political purpose.

In the revised manuscript, we have

“Finally, to increase the response rate and obtain respondents' truthful opinions, we collected the data using a face-to-face survey. During the survey, one enumerator took 1-2 hours to complete one questionnaire, and each survey enumerator completed 3-5 questionnaires per day. To ensure the authenticity of the survey，we randomly selected respondents in the sample villages. Because our work focused on the income effect of the land rental market on land renting-in households and their corresponding renting-out households, when we interviewed the renting-out households, we tried to identify their matching renting-in households by the land lease contract. To estimate the land rental market's income effect, we also interviewed farmers who were not participating in the land rental market in the sample villages, which was used as a control group. ” (page 10)

**Participation: The definition of participation as given by the authors is clear enough. However, the definition is silent about the time frame that a household should participate to be considered as “Participant”. This is important for attribution.**

**Reply**: Thanks for the comments. In our survey, the time frame of participants was determined through lease contracts. Most of the contract was between 8-20 years. However, the current study mainly focuses on the welfare effect of participation in the land market rather than the effect of contract length. Future research can investigate the impact of land rent-in/rent-out contract length on household welfare.

In the revised manuscript, we stated “Moreover, most of the land lease agreement was between 8-20 years. Because the current study mainly focuses on the welfare effect of participation in the land market, we did not examine the impact of lease agreement length on the land rental market participants’ income. Future research can investigate the effect of land rent-in/rent-out contract length on household welfare.” (page 11)

**Endogeneity. The current methodology used focuses on the selectivity bias. However, the authors raised the issue of bias due to unobservable factors. Some tests need to be performed to clarify whether or not these factors create the problem of endogeneity and if yes propose a way to deal with the problem.**

**Reply**: Thanks for the comment. The main reason that we used PSM in the current research is to avoid the endogeneity issues that may be associated with regression analysis. Thus, we are not quite sure what you mean by “these factors create the problem of endogeneity.” If you can provide any reference for us to check, it would be really appreciated.

PSM may be sensitive to “unobservable factors” that are not included in the covariates when we did the matching. To make sure our results are robust and will not be affected by the unobservable factors, we conducted a sensitivity analysis and reported additional results in Table 6. Table 6 added two columns to show the p-values at different levels of gamma values (1.0 and 0.5). The results show that our conclusions are insensitive to the unobservable factors, therefore robust.

**Discussion and policy relevance: How do these results fit into the existing literature in this area? There was no effort by the authors to compare the results obtained in the study with the one in the literature. What is the policy relevance of this research? The current analysis supposes that participating in farmland renting market affect household the same way which is not the case in practice. It would be good to apply some decomposition perhaps by some socioeconomics characteristics such as gender to appreciate that difference.**

**Reply:** we separate your comments and will reply to them one by one.

1. **How do these results fit into the existing literature in this area? There was no effort by the authors to compare the results obtained in the study with the one in the literature.**

**Reply**: In the introduction, the authors the existing literature mainly focused on efficiency, equity, and welfare. The impact on welfare, and the existing literature is discussed separately from renting out households and renting in households.

2. **What is the policy relevance of this research? The current analysis supposes that participating in farmland renting market affect household the same way which is not the case in practice.**

**Reply:** This research wants to solve the question about how the rental market improve income in the smallholder farming system for both renting-in and renting-out household. Our results show that for renting-in households, joining the rural land rental market can increase farmers' welfare, which is consistent with the previous research conclusions(Fei Chen,2015; Min Shi ,2017). While from the perspective of renting-out households, joining the rural land rental market reduces the welfare of farmers, which is inconsistent with the previous research conclusion. This may be related to the lagging effect of land system reform. So our research does not assume that “participating in farmland renting market affect household the same way”. And our research is an extension of the existing literature in this area. So policy relevance is, participation in the farmland rental market significantly increases the income of renting-in households, while it decreases the income of renting-out households, which might result from the lag effect of the land system reform temporarily.

3. **It would be good to apply some decomposition perhaps by some socioeconomics characteristics such as gender to appreciate that difference**

Thanks for the comments. This is a good suggestion. However, we could not separate the samples by gender and do the PSM to see whether the welfare of participation differs by gender or not. The reason is that we required the interviewee to be a person who is familiar with agricultural production. Although both husband and wife in rural China make joint decisions most of the time, males are mainly responsible for agricultural production and therefore are more familiar with the land rental issues. For instance, males account for 97% of the sample in this study. Therefore, it is hard to divide the samples by gender. With more samples collected, we could try to differentiate the welfare effect of the farmland renting market participation by some demographics or farm characteristics (e.g., farm land size). We added this as a future research directions at the end of the paper.

“Subsequent studies can expand the sample areas with different levels of economic development and carry out research based on larger sample data. With more data collected, the analyses can be conducted for farmers with different land sizes, which can provide a deep insight into the effect of farmland rental markets on the income of households with various farm sizes.” (page 24)

**Finally, the paper would benefit from some additional editing to ensure that concepts and terminology are stated clearly. Some of my edits are in the attachment.**

**Reply**: We appreciate your edits and comments. We also go through the paper thoroughly to make sure that all the concepts and terminologies are stated clearly.
