# Supplementary material for: How do farmland rental markets affect farmers’ income? Evidence from a matched renting-in and renting-out household survey in Northeast China
Source: PLoS One. 2021 Sep 30;16(9):e0256590. doi: 10.1371/journal.pone.0256590 (PMC8483330; doi:10.1371/journal.pone.0256590)
Supplement: Attachment — Submitted filename: PONE_Anonymous Referee Report.docx [file pone.0256590.s002.docx]

**Anonymous Referee Report**

Topic: How do Farmland Rental Market Affect Farmers’ Income: Evidence from a Matched Renting-in and Renting-out Household Survey in Northeast China

**Summary (Editor):**

The study used a farm household survey of 810 rural households in China to examine the factors influencing participation in the farmland rental market. The study also evaluated the impact of the rental market on farmers’ income. Results of the study show that household off-farm income, family members’ part-time employment, and agricultural subsidies, agricultural cooperatives significantly affect farmers’ participation in the farmland rental market. Finally, participation in the farmland rental market increases the income of renting-out households.

Generally, the subject of investigation is relevant within the context of land rental market. I find the research interesting especially within the context of developing countries where the issues of the welfare effect of renting-out households in the rental market are often ignored. The methodology employed for the study is relevant and adequately addressed the research questions. The conclusion of the study is adequately supported by the findings. However, the paper will improve significantly if the major and minor comments are adequately addressed. I recommend a **MAJOR revision**.

**Major Comments**

1. The author(s) should show the graph indicating the region of common support.
2. In the Rosenbaum sensitivity test you conducted; it will be appropriate to show the performance of the estimates at the different levels of gamma values rather than showing only the gamma value of 1.5.
3. The discussion of the results must be strengthened.
4. Differential analysis - an additional suggested analysis could be categorizing the farmers (rent-in and rent-out) into different land size (terciles) and perform the PSM. This will allow the author(s) to establish the sensitivity of their results based on changes in the land size.

**Minor Comments**

1. There are some grammatical errors and spelling mistakes that might have escaped the attention of the author(s) – For example page 15, 16, 17, 25
2. The mathematical notations of the variables must be clear and precise.
3. Use formal writing style. E.g. use “cannot” instead of “can’t”.
4. The tables must be properly formatted.
5. I recommend a thorough formatting of the equations and the text as well.
